# Cascading effects of soil water content on leaf chemistry and damage: implications for plant susceptibility to pests and pathogens in a drier world

**DOI:** 10.3389/fpls.2026.1914073

**Published:** 2026-10-20

**Authors:** Álvaro Gaytán, Luis Matías, Óscar Godoy, Ignacio M. Pérez-Ramos, Pablo Homet, Xoaquín Moreira, Lorena Gómez-Aparicio

**Affiliations:** 1 Instituto de Recursos Naturales y Agrobiología de Sevilla, Consejo Superior de Investigaciones Científicas (IRNAS-CSIC), Sevilla, Spain; 2 Departamento de Biología Vegetal y Ecología, Universidad de Sevilla, Sevilla, Spain; 3 Estación Biológica de Doñana, Consejo Superior de Investigaciones Científicas (EBD-CSIC), Sevilla, Spain; 4 Misión Biológica de Galicia, Consejo Superior de Investigaciones Científicas (MBG-CSIC), Pontevedra, Galicia, Spain

**Keywords:** foliar pathogens, free feeding herbivory, global change, Mediterranean mixed forests, non-structural carbohydrates, phenolics, soil water content, tannins

## Abstract

Climate change is causing major alterations in precipitation regimes and soil water availability, with far-reaching effects on plant traits. Changes in plant chemistry under drought scenarios are particularly relevant, as they can modulate plant interactions with antagonistic organisms. However, how such changes in plant chemistry affect plant susceptibility to biotic damage remains poorly understood. Here we integrated results from a systematic literature review and a field study in Mediterranean mixed forests to advance our understanding of the cascading effects of reduced soil water content on leaf reserves, defences and damage caused by herbivores and pathogens. The literature review revealed that studies linking soil water content with leaf chemistry and damage remain scarce and reported a variety of direct and indirect relationships between the three components. Nevertheless, most studies found higher leaf damage under drier conditions. Results from our field study also supported that leaf damage increased with decreasing soil water content, a relationship largely mediated by changes in leaf phenolic compounds. In Mediterranean forests, phenolics play a major role in protection against desiccation and UV radiation due to their antioxidant properties, yet certain phenolic compounds can also benefit antagonists. Overall, our findings suggest that leaf damage can increase in a drier climate, forcing tree species to face fundamental trade-offs when protecting themselves from the combined stress imposed by abiotic (drought) and biotic (pests and pathogens) components of global change.

## 1 Introduction

Since the mid-20th century, climate change has been exerting significant effects on the functioning of terrestrial ecosystems (Stocker et al., 2013). One of its primary components is the alteration of precipitation regimes, which involves significant rainfall reductions in many parts of the world (Trenberth, 2011; Balting et al., 2021). Such rainfall reduction is predicted to intensify in both frequency and severity over the coming decades (Seneviratne et al., 2023), resulting in decreased soil water availability with far-reaching effects on plant phenotypes (Brando et al., 2008; Liu et al., 2015; Bashir et al., 2021). Among plant traits, changes in plant chemistry are particularly relevant under drought scenarios. This is because such changes can modulate plant resistance to environmental stress and biotic interactions with antagonistic species (Mody et al., 2009; Gutbrodt et al., 2012a; Kuczyk et al., 2021; Ganthaler et al., 2023). More specifically, antagonistic interactions involving pests and pathogens have the potential to cause strong reductions in plant growth, reproduction and fitness (Zvereva and Kozlov, 2014; Nazarov et al., 2020). Yet, how changes in plant chemistry driven by altered soil water content can indirectly influence plant susceptibility to biotic damage remains poorly understood. Understanding these bottom-up effects is crucial for forecasting plant susceptibility to emergent pests and pathogens under future drought scenarios.

Drought triggers physiological plant responses that can translate into significant alterations in their chemistry (Moradi, 2016; Zandalinas et al., 2018; Ali et al., 2025). These changes often reflect adaptive strategies to cope with water scarcity (Selmar and Kleinwächter, 2013; Orians et al., 2019). In response to drought, plants generally close their stomata to minimize water loss through evapotranspiration (Morgan, 1984; Limousin et al., 2009; Quentin et al., 2012; Liu et al., 2025). Stomatal closure leads to a decrease in photosynthetic activity (C-uptake) and, consequently, to a reduction in the levels of non-structural carbohydrates, which are the primary products of photosynthesis in plants (Rosas et al., 2013; Guo et al., 2022). A low availability of carbohydrates can further induce trade-offs among the different C-sinks related with the primary and secondary metabolism of plants, resulting in many cases in a lower allocation of substrates to the synthesis of defensive compounds (Lemmermeyer et al., 2015; Hartmann and Trumbore, 2016; Gessler and Grossiord, 2019). However, some studies have also reported increases in the levels of carbohydrates and defensive compounds in response to drought, because these compounds also participate in plant strategies to cope with water deficit (Hale et al., 2005; Rivas‐Ubach et al., 2014; Pineda et al., 2016). Non-structural carbohydrates, mainly sugars and starch, are involved in osmoregulation and accumulate under drought to maintain leaf turgor and regulate water potential (Dietze et al., 2014). Similarly, some defensive compounds (e.g. phenolic compounds) are accumulated by plants to mitigate the detrimental effects of drought stress, providing antioxidant properties, UV protection, and desiccation tolerance (Varela et al., 2016; Jansen et al., 2022; Park et al., 2023; Shoaib et al., 2023). Because of the complex responses that water deficits can induce in plant chemistry, it remains uncertain how climate-change related drought might alter levels of non-structural carbohydrates and defensive compounds in plant species.

Alterations in plant chemistry induced by limited soil water availability can cascade to alter levels of biotic damage aboveground in a bottom-up pathway (Gutbrodt et al., 2011; Rivas‐Ubach et al., 2014; Lin et al., 2021; Ganthaler et al., 2023). Bottom-up pathways occur when a lower trophic level (plants) affects upper trophic levels (e.g. herbivore insects and pathogens) by means of resource restriction (e.g. drought) (White, 1978). The mechanisms underlying these pathways are multiple and can result in different outcomes. On the one hand (scenario 1; **Figure 1a**), when low soil water content limits C fixation, leading to a decrease of non-structural carbohydrates and defensive compounds, plants may become more susceptible to herbivores and pathogens due to weakened defences, resulting in higher leaf damage and leaf loss (Gutbrodt et al., 2012a; Heyman et al., 2021). On the other hand (scenario 2; **Figure 1b**), if low soil water content stimulates the accumulation of compounds with a dual protective role against both abiotic and biotic stresses (e.g. phenolics), then plants may become less palatable due to reduced nutritional quality, experiencing less leaf damage by herbivores and pathogens (Lin et al., 2021). Nevertheless (scenario 3; **Figure 1c**), it is also possible that certain types of carbohydrates and defensive compounds accumulated in drought-stressed plants because of their osmoregulatory and antioxidant properties act as attractors of antagonists, stimulating leaf damage (Gutbrodt et al., 2012a; Rivas‐Ubach et al., 2014). Understanding under which circumstances each of these alternative scenarios is more likely to occur is crucial for predicting how susceptible plant species and ecosystems are to altered levels of soil water availability and biotic damage under climate change.

**Figure 1:**
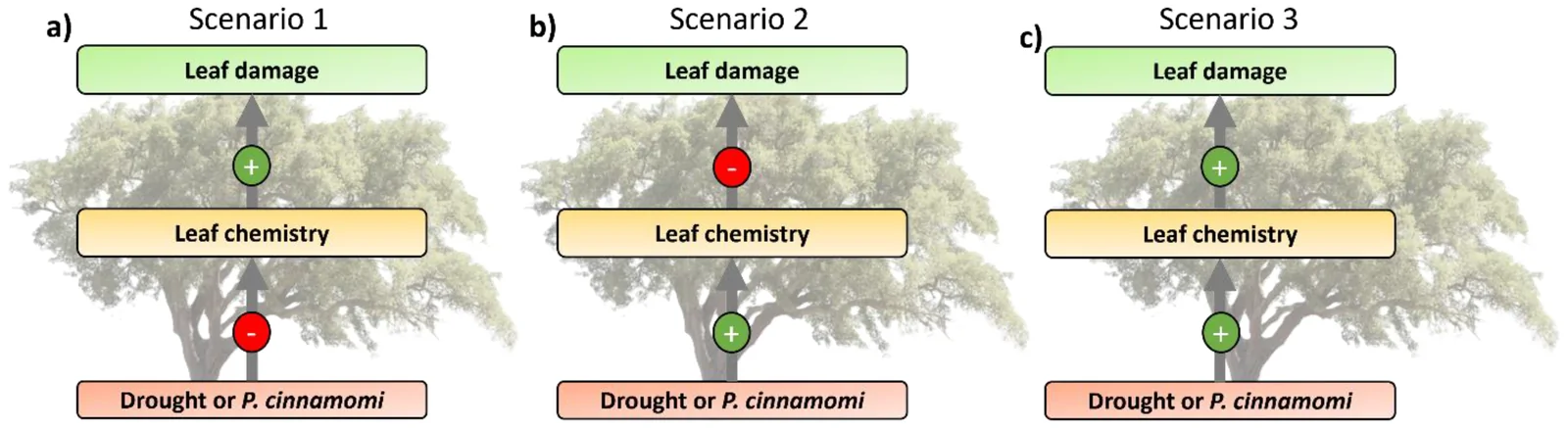
Scenarios showing different potential cascading effects of drought- or *Phytophthora*-induced changes in leaf chemistry on leaf damage. **(a)** Scenario 1: drought- or *Phytophthora*-induced reductions in chemical compounds promote leaf damage; **(b)** Scenario 2: drought- or *Phytophthora*-induced increases in chemical compounds reduce leaf damage; and **(c)** Scenario 3: drought- or *Phytophthora*-induced increases in chemical compounds increase leaf damage.

In this study, we aimed to advance our understanding of the cascading effects of decreasing soil water availability in the context of climate change on leaf chemistry (non-structural carbohydrates and defences) and leaf damage caused by herbivores and pathogens using a dual yet complementary approach. First, we did a systematic review of studies assessing the impact of drought on leaf chemistry (non-structural carbohydrates and/or defensive compounds) and damage by herbivores and pathogens to evaluate the state-of-the-art and the existing support for the alternative scenarios linking soil water content, plant chemistry and leaf damage. Second, we tested such linkages in the field taking advantage of a gradient in soil water content amplified by a rainfall reduction infrastructure installed in Mediterranean mixed forests invaded by the soil-borne pathogen *Phytophthora cinnamomi*, one of the most aggressive exotic pathogens on earth (Lowe et al., 2000). For two years, we measured leaf non-structural carbohydrates (sugars, starch; “reserves” hereafter) and chemical defences (total phenolics and condensed tannins) in three dominant tree species (*Quercus suber*, *Quercus canariensis* and *Olea europaea*), as well as the level of leaf damage by free feeding herbivores and pathogens. With our field study, we aimed to answer three specific questions: 1) Does a reduction in soil water content lead to lower concentrations of leaf reserves and defences in adult trees? We hypothesized that in Mediterranean-type ecosystems, where water is the main limiting factor for plant performance, a reduction in soil water levels could restrict C assimilation, resulting in reduced concentrations of reserves and defences (Rosas et al., 2013; Dickman et al., 2015; Guo et al., 2022); 2) Does a reduction in soil water content induce changes in leaf reserves and defences that alter leaf damage by herbivores and pathogens? We expected that a decrease in leaf reserves and defences due to lower soil water content would translate into higher leaf damage (scenario 1, **Figure 1**) (Rivas‐Ubach et al., 2014); and 3) Can the soil-borne pathogen *P. cinnamomi* induce bottom-up effects on leaf chemistry and damage similar to those caused by reduced soil water content? Because *P. cinnamomi* destroys fine roots and limits water uptake (Hardham, 2005), we expected this biotic stressor to trigger indirect effects on leaf damage similar to those induced by soil water deficits. The answers to these questions will provide new insights into how global change drivers such as increasing drought and exotic soil-borne pathogens might alter damage levels by aboveground antagonists in forests located in one of the most vulnerable biomes (the Mediterranean) to climate change (Newbold et al., 2020).

## 2 Methods

### 2.1 Literature review

We carried out a systematic search of the existing studies analysing the impact of drought-induced changes in leaf reserves (= non-structural carbohydrates) and chemical defences (e.g., phenolics, alkaloids, glucosinolates) on leaf damage by free feeding herbivores and pathogens. We searched in Web of Science on 2 September 2025 using the following structure of terms and boolean operators: (*drought OR soil water content OR soil water availability OR soil water scarcity OR soil water deficit OR water stress*) AND (chemistry OR defense* OR defence* OR reserve* OR carbohydrate* OR secondary compound*) AND (leaf OR leaves OR needle*) AND (damage OR attack OR herbivor* OR folivor* OR pathogen*). With this search, we excluded studies that addressed only isolated links (drought-leaf chemistry, leaf chemistry-leaf damage), as our focus was on the cascading effects of soil water content on leaf damage, mediated by leaf chemistry.

Our literature search yielded 3214 studies (identification; **Appendix S1**; **Supplementary Figure S1**). We first removed reviews, proceeding papers, book chapters and theses (screening; **Appendix S1**; **Supplementary Figure S1**). From the 2511 remaining studies, a study was considered valid for the review if it fulfilled the following specific criteria (eligibility; **Appendix S1**; **Supplementary Figure S1**). Regarding soil water content, we focused on studies where plants under different levels of soil water content were exposed simultaneously to the same herbivore/pathogen community. Therefore, we discarded observational studies comparing plants in wet vs. dry sites or seasons. Regarding leaf chemistry, studies should focus on non-structural carbohydrates (sugar and starch; Hansen and Møller, 1975) and secondary metabolites involved in direct defences against leaf damage (*sensu* Fernández et al., 2024). Thus, we discarded studies that analysed secondary metabolites involved in indirect defences against leaf damage (e.g., volatiles), as well as hormones involved in the signalling of anti-herbivory defence regulation (e.g., abcisic or jasmonic acid). Regarding leaf damage, studies should focus on folivorous herbivores, which consume leaf tissue (i.e., leaf chewing insects, skeletonizers and leaf miners). Thus, we discarded studies focusing on other herbivore guilds (e.g., sap-sucking insects, gall inducers) whose damage is difficult to estimate, as well as studies that applied artificial damage. We also discarded studies that did not measure leaf damage, but insect preference or performance. Only ten studies met the selection criteria (**Appendix S1**; **Supplementary Figure S1**).

### 2.2 Study sites

The study sites are located in Los Alcornocales Natural Park (Cádiz, Spain, 36.568749, −5.535640, **Appendix S1**; **Supplementary Figures S2a, b**). Los Alcornocales is the largest and best-preserved *Quercus suber* (L.) forest in Europe (Urbieta et al., 2008). The climate is sub-humid Mediterranean and 95% rainfall is concentrated from October to May, ranging from 620 to 2100 mm (Junta de Andalucía, 2017). Soils are sandy, acidic and poor in macronutrients, derived from bedrock dominated by Oligo–Miocene sandstones. We selected two study sites separated by *c*. 4 km representative of the main mixed oak forests of the area. One site was a closed forest in wet areas with sandy soils, where *Quercus suber* co-exist with the shade-tolerant *Quercus canariensis* (Willd.). The other site was an open mixed forest common in drier areas of the Natural Park with clayish soils dominated by *Q. suber* and the drought-tolerant *Olea europaea* var. *sylvestris* (L.). *Q. suber* and *O. europaea* are evergreen species with a leaf phenology characterized by the production of new leaves during the late spring (May-June), coinciding with a peak of litterfall that in *Q. suber* can represent most of the leaf biomass produced in the previous year (Caritat et al., 1996). In contrast, *Q. canariensis* is a semi-deciduous species in which leaves abscission occurs along the winter months, with old leaves co-occurring with new leaves during a few weeks in early spring (García-Mijangos et al., 2015; Loidi, 2017).

The two study forests are invaded by the exotic soil-borne pathogen *Phytophthora cinnamomi* (Serrano et al., 2024). This pathogen was first isolated in Los Alcornocales in the early 1990s (Brasier, 1992), and since then it has been identified as the main driver of *Quercus* spp. decline in the Iberian Peninsula and southern Europe (Camilo-Alves et al., 2013; Matías et al., 2019). *P. cinnamomi* is a generalist pathogen with a wide range of hosts that strongly differ in susceptibility. For example, among the three study species, *Q. suber* is much more susceptible to the pathogen than the coexisting *O. europaea* and *Q. canariensis* (Gómez et al., 2020). The life cycle of *P. cinnamomi* is favoured under conditions of free water in the soil, which allows for spore dispersion and root infection (Hardham and Blackman, 2018). Consequently, it shows a strong spatial and temporal variability in the forest soil with large inoculum densities being associated with the presence of high-quality hosts (e.g. *Q. suber*) and high soil water availability (Gómez‐Aparicio et al., 2012; Serrano et al., 2022).

In Mediterranean forests, a wide variety of herbivorous insects and foliar pathogens attack tree leaves (Southwood, 1961; Gillett-Kaufman et al., 2021; Hernández‐Agüero et al., 2022; Gaytán, 2026a). The community of free feeding herbivores is primarily composed of lepidopteran species such as *Lymantria dispar* or *Tortrix viridana* (Gaytán et al., 2018; Hernández‐Agüero et al., 2022). The peak of herbivory occurs in spring, but herbivores can consume leaves until the end of the growing season, barring periods of extreme heat (Tammaru et al., 2001; Gripenberg et al., 2007; Gaytán et al., 2018). Foliar pathogens, predominantly fungal species such as *Cystodendron* spp. or *Spilocaea* spp (Obanor et al., 2008; Moricca et al., 2016), cause most lesions during spring, a period characterized by the highest spore loads and the highest leaf damage (Materatski et al., 2019; Gaytán et al., 2022).

### 2.3 Rainfall exclusion infrastructures

In March 2016, rainfall exclusion infrastructures were installed in the two forest types with the aim to reduce soil water availability and evaluate its effect on the dynamics of Mediterranean mixed forests. In each of the two study sites, we established six plots of 20 × 15 m distributed in three blocks (i.e. 2 plots per block), with an average distance of 30 m among blocks. All plots were fenced to avoid the interference of large ungulates. In each block, one of the plots was assigned to the rainfall exclusion treatment, whereas the other plot served as control. The rainfall exclusion treatment was designed to reduce in 30% the amount of rainfall, reproducing future climate change scenarios for southern Spain (model CMIP5 for the scenario RCP 8.5 for the period 2081-2100; Stocker et al., 2013). We used polyvinyl chloride (PVC) gutters to cover 30% of the plot area excluding a similar amount of rainfall (cf Homet et al., 2021), placed >1 m height over the forest soil (**Appendix S1**; **Supplementary Figure S2c**). In control plots, the same gutters were placed upside down to simulate potential secondary effects of the infrastructure on the forest microenvironment maintaining the natural precipitation regime. We individually marked all trees within the plots (3–4 trees per plot and species), excluding those in a buffer area of 1 m to avoid edge effects. Overall, the field study included 21 *Q. suber* and 22 *Q. canariensis* trees in the closed forest and 21 *Q. suber* and 22 *O. europaea* trees in the open forest (n = 86 trees in total). For more details of the rainfall exclusion infrastructure installation and design, see Homet et al. (2021).

### 2.4 Quantification of soil water content and *P. cinnamomi* abundance

For monitoring soil water content in the study plots, we used a FDR sensor (PR2 Profile Probe, Delta-T Devices, UK) that takes measurements at four depths (10, 20, 30 and 40 cm). Measurements were conducted monthly at six points per plot where access tubes were permanently installed for easy probe insertion and removal. The rainfall reduction infrastructure significantly reduced soil water content at the forest sites during the study period, particularly at greater depths (see Homet et al., 2021; Gaytán et al., 2024). The largest differences occurred after strong rainfall events, as those occurred during the spring of 2018, where soil moisture reached values 33% higher in control than in drought plots (**Appendix S1**; **Supplementary Figures S3, S4**). For this study, we used the measurements of the four depths to calculate a mean value for the 0–40 cm profile at each measurement date. Then, we calculated the average soil water content in spring (February, March and April) and summer (July, August and September) of the two study years (2017 and 2018). Spring matches the optimal climatic conditions for plant growth in Mediterranean areas (wet season), whereas summer matches the moment of maximum water limitation (dry season).

The abundance of *P. cinnamomi* inoculum in the soil was quantified in spring of the two study years, matching with the population peak of the pathogen in the study area (Serrano et al., 2022). We followed the methodology described in Serrano et al. (2024). Briefly, soil samples (5–20 cm) were collected within 1 m of each tree trunk using a soil auger. In the lab, soils were air dried and sieved through a 2 mm mesh. Each sample was used to prepare a soil-water agar mix that was placed on Petri dishes (20 per sample) containing NARPH (Hüberli et al., 2000). Petri dishes were incubated in the dark, and the colonies of *P. cinnamomi* growing on the plates were morphologically identified under the inverted microscope and counted. As shown in Serrano et al. (2024), the rainfall exclusion infrastructure had a negative effect on pathogen abundance across sites and species, particularly in wet years such as 2018.

### 2.5 Quantification of leaf chemistry and damage

To quantify leaf chemistry and damage by free feeding herbivores and foliar pathogens, we collected leaf samples of each of the 86 target trees twice per year, in April (early spring) and September (late summer) of 2017 and 2018. Leaves collected in early spring (i.e., before new leaves are totally unfolded) represent “old” leaves formed in the previous growing season, and therefore their damage levels represent the accumulated damage suffered during the previous year. On the contrary, leaves collected at the end of the summer represent “new” leaves from the current year, so their damage levels represent the attack suffered only during the spring and summer seasons of the current year. Three branches were cut per tree using an extendable pole at approx. 6 m height in the outer area of the canopy. Branches were introduced into a plastic bag to maintain moisture and were transported into an ice box to the laboratory, where they were stored at -20° C. In the laboratory, a representative subsample of 30 leaves per tree was oven-dried at 45 °C during 48 h and ground with liquid nitrogen and used for extraction of non-structural carbohydrates (NSC = soluble sugars and starch), total phenolics and condensed tannins. Non-structural carbohydrates were quantified using the anthrone method in 20 mg of dry tissue (cf. Hansen and Møller, 1975), while total phenolics and condensed tannins were quantified colorimetrically by the Folin-Ciocalteu assay in 300 mg of dry tissue (cf. Moreira et al., 2009). We focused on phenolics because they are the primary constitutive and inducible defences in our tree species (Galmán et al., 2019). Since condensed tannins are particularly relevant chemical defences of *Quercus* spp. against leaf damage due to their high toxicity, we quantified and analysed them separately from the pool of total phenolics (Harding, 2019).

A second subsample of 30 leaves per tree was used for estimation of leaf damage. For each leaf, we visually estimated the percentage of herbivory following a well-established class system with eight categories: 0%, 1%-5%, 6%-10%, 11%-15, 16%-25%, 26%-50%, 51%-75% and 76%-100% (cf. Castagneyrol et al., 2013). We calculated herbivory at tree level by averaging the midpoints of each category across tree leaves (cf. Gaytán et al., 2022). Herbivory levels represented mostly the consumption of leaf tissue by folivorous insects (leaf chewing insects; **Appendix S1**; **Supplementary Figure S5**), since other types of leaf herbivory such as leaf miners or gallers were virtually absent from our samples (< 5% of the used leaves). To quantify damage by pathogens, leaves were scanned and the percentage of leaf surface damaged by pathogens (i.e. necrosis in different colours and forms, **Appendix S1**; **Supplementary Figure S5**) was measured using the image-processing software *ImageJ* (Schneider et al., 2012). To avoid biases, all leaves were measured by a single person following the standards described in **Appendix S1**; **Supplementary Figure S5**.

### 2.6 Statistical analysis

We used General Linear Mixed Models (GLMMs) to explore the bilateral relationships between soil water content (SWC) or *P. cinnamomi* abundance (predictors) and leaf chemistry (response variable; Equation 1), as well as among leaf chemistry (predictor) and leaf damage (response variable; Equation 2). Models were fitted separately for each combination of season (spring or summer), site (closed forest or open woodland), species (*Q. suber* or *Q. canariensis* in the closed forest, *Q. suber* or *O. europaea* in the open woodland), chemical compound (non-structural carbohydrates, total phenolics or condensed tannins), and type of attacker (herbivores or pathogens). Data from the two study years (2017 and 2018) were pooled together to generalize our results and increase the number of observations, so tree identity was included as a random factor to account for repeated measurements. Block was included as a covariate and plot as a random factor to control for the nested structure of the study design. Leaf chemistry was modelled using a Gaussian distribution of errors, whereas leaf damage was modelled with a beta-error distribution because it was bounded between 0 and 100. Models were constructed using the R-packages *lme4* and *glmmTMB* (Bates et al., 2015; Brooks et al., 2023). We assessed model fit using the diagnostic tools in the *DHARMa* R-package (Hartig, 2020) and the variance explained by the models was calculated using the function *‘r.squaredGLMM’* from the R-package *MuMIn* (Barton, 2009). We also used GLMMs to explore differences in leaf compounds (non-structural carbohydrates, phenolics and condensed tannins) and leaf damage (herbivory and pathogen infection) between seasons (spring and late summer), including block as a covariate and tree as a random factor.

(1) $$Y=\beta 0+\beta 1_{Block}+\beta 2_{SWC}+\beta 3_{P.\;cinnamomi}+µ1_{Plot}+µ2_{Tree}+\epsilon$$

(2) $$Y=\beta 0+\beta 1_{Block}+\beta 2_{Leaf\;chemistry}+µ1_{Plot}+µ2_{Tree}+\epsilon$$

We used Structural Equation Models (SEMs) to model bottom-up direct and indirect effects of soil properties representative of global change stressors (soil water content and *P. cinnamomi* abundance) on damage by free feeding herbivores and foliar pathogens mediated by alterations in non-structural carbohydrates and defensive compounds. Piece-wise SEM were constructed separately for each combination of season, species and site using the function ‘*psem’* in the R-package *piecewiseSEM* (Lefcheck, 2016). In all models, we added tree identity as a random factor to account for repeated measurements on the same individual tree among years. The structure of our SEMs and the rationale of their paths is detailed in **Appendix S1**; **Supplementary Figure S6**. Goodness of fit of SEMs was evaluated with Fisher’s C-test (Gaytán, 2026b). Direct, indirect and total associations were estimated as standardised partial regression coefficients using the R-package *semEff* (Murphy, 2022). All statistical analyses were conducted with R software version 4.1.1 (R Development Core Team, 2024).

## 3 Results

### 3.1 Literature review

Only ten studies (representing 15 case studies) met the selection criteria of our literature review (**Table 1**), reflecting a knowledge gap in the literature. Eight were conducted under controlled conditions in the greenhouse, whereas only two were conducted under more natural conditions (field or common garden, **Table 1**). The studies were heterogeneous in their focal plant groups, including four on tree species, one on a shrub species, and five on herbaceous species (**Table 1**). Nine studies reported data on leaf chemical defences, three on leaf reserves, and two on both (**Table 1**). Overall, these studies yielded 15 plant-attacker-compound combinations (hereafter case studies). Of these, two assessed leaf damage by pathogens (Enright and Cipollini, 2011; Ganthaler et al., 2023), whereas the rest focused on leaf damage by herbivores (**Table 1**). Among the case studies, about 33% of them (5/15) did not find drought effects on leaf chemistry (**Table 1**). Of the remaining ten that did detect drought effects, two case studies (Gutbrodt et al., 2011, 2012a) supported scenario 1 (i.e. drought-induced reductions in chemical compounds promote leaf damage, **Figure 1**), two case studies (Enright and Cipollini, 2011; Lin et al., 2021) supported scenario 2 (i.e. drought-induced increases in chemical compounds reduce leaf damage), and four case studies (Gutbrodt et al., 2012a; Prill et al., 2014; Rivas‐Ubach et al., 2014) supported scenario 3 (i.e. drought-induced increases in chemical compounds increase leaf damage). Only one case study (Gutbrodt et al., 2011) reported a pattern not captured by our three scenarios, where drought-induced reductions in leaf defences of *Alliaria petiolata* were associated with lower damage by the lepidopteran *Pieris brassicae* (**Table 1**). Overall, 9 out of 15 case studies found higher leaf damage under drought than under control soil water conditions (**Table 1**).

**Table 1:** Summary of studies assessing the impact of drought-induced changes in leaf chemistry (reserves and defences) on leaf damage by free feeding herbivores and pathogens.

Case study	Study	Study type	Plant			Attacker			Drought effects		
			Species	Life form	Origin	Species	Type	Chemical group	Leaf chemistry	Leaf damage	Scenario
1	Bisigato et al., 2015	Greenhouse	*Larrea divaricata*	Shrub	Argentina Argentina	*Nyctelia circumundata* *Nyctelia circumundata*	Herbivore	Defences	0	0	
2		Greenhouse	*Prosopis alpataco*	Shrub			Herbivore	Defences	0	0	
3	Enright and Cipollini, 2011	Greenhouse	*Alliaria petiolata*	Herbaceous	USA	*Erysiphe alphitoides*	Pathogen	Reserves	+	-	2
4	Ganthaler et al., 2023	Greenhouse	*Picea abies*	Tree	Austria	*Chrysomyxa rhododendri*	Pathogen	Defences	0	+	
5	Gutbrodt et al., 2011	Greenhouse	*Alliaria petiolata*	Herbaceous	Germany	*Pieris brassicae*	Herbivore	Defences	-	-	NA
6		Greenhouse	*Alliaria petiolate*	Herbaceous	Switzerland	*Spodoptera littoralis*	Herbivore	Defences	-	**+**	1
7	Glynn et al., 2004	Greenhouse	*Salix* sp.	Tree	Sweden	*Phratora vulgatissima*	Herbivore	Defences	-	0	
8	Gutbrodt et al., 2012a	Greenhouse	*Malus domestica*	Tree	Switzerland	*Spodoptera litorallis*	Herbivore	Reserves	+	**+**	3
9		Greenhouse	*Malus domestica*	Tree	Switzerland	*Spodoptera litorallis*	Herbivore	Defences	-	**+**	1
10	Gutbrodt et al., 2012b	Greenhouse	*Brassica oleracea*	Herbaceous	Switzerland	*Pieris brassicae*	Herbivore	Defences	0	**+**	
11		Greenhouse	*Brassica oleracea*	Herbaceous	Switzerland	*Spodoptera littoralis*	Herbivore	Defences	0	**+**	
12	Lin et al., 2021	Greenhouse	*Solanum lycopersicum*	Herbaceous	United States	*Manduca sexta*	Herbivore	Defences	+	**-**	2
13	Prill et al., 2014	Common garden	*Brassica nigra*	Herbaceous	United Kingdom	*Psylliodes chrysocephalus*	Herbivore	Defences	+	**+**	3
14	Rivas‐Ubach et al., 2014	Field	*Quercus ilex*	Tree	Spain	Full community	Herbivore	Reserves	+	+	3
15		Field	*Quercus ilex*	Tree	Spain	Full community	Herbivore	Defences	+	+	3

Positive (+), negative (-), and neutral (0) symbols indicate whether leaf chemistry and leaf damage were higher, lower, or unchanged under drought compared to control conditions. For those case studies reporting cascading effects of drought on leaf chemistry and damage, the theoretical scenario supported (1: -+, 2: +-, 3: ++; **Figure 1**) is also indicated.

Scenario 1: drought-induced reductions in chemical compounds promote leaf damage; Scenario 2: drought-induced increases in chemical compounds reduce leaf damage; Scenario 3: drought-induced increases in chemical compounds increase leaf damage; NA, pattern not fitting any of the defined scenarios.

Green indicates positive effect, red indicate negative effect and grey indicate null effect.

### 3.2 Field study

The rainfall exclusion infrastructure significantly reduced soil water content at the forest sites during the study period (**Appendix S1**; **Supplementary Figure S7**). The rainfall reduction amplified the gradient of soil water content in the study sites towards drier values, particularly during spring (**Appendix S1**; **Supplementary Figures S8, S9**).

Overall, defence levels were higher in summer leaves than in spring leaves, while the opposite pattern was observed for leaf reserves (**Appendix S1**; **Supplementary Figure S10**; **Supplementary Table S2**). Soil water content affected leaf chemistry of the three species studied (*Quercus suber*, *Q. canariensis*, *Olea europaea*), at both the closed forest (**Appendix S1**; **Supplementary Figure S8**) and the open woodland (**Supplementary Figure S9**). These effects were, however, much more common for chemical defences than for non-structural carbohydrates, total leaf phenolics decreasing with increasing soil water content in most sites and species combinations (**Appendix S1**; **Supplementary Figures S8, S9**). Such negative relationships were detected for all species in summer samples, whereas in spring samples they appeared only for the semideciduous species *Q. canariensis* (**Appendix S1**; **Supplementary Figures S8, S9**). Regarding relationships between abundance of *P. cinnamomi* in the soil and leaf chemistry, models rarely supported significant effects (**Appendix S1**: **Supplementary Figures S11, S12**). Interestingly, defence compounds (total phenolics) of *Q. suber* leaves in the closed forest showed a significant increase with the abundance of the pathogen in the soil (**Appendix S1**; **Supplementary Figure S11**).

The magnitude of leaf damage was highly variable among trees of the same species and site. Both damage by herbivores and pathogens peaked at 40-50% for *Quercus* species at both the closed forest (**Appendix S1**; **Supplementary Figure S13**, **S14**) and the open woodland (**Appendix S1**; **Supplementary Figure S15**), whereas in *Olea* trees rarely reached 10% (**Supplementary Figure S16**). Herbivore damage was similar in spring and summer samples, while pathogen damage was generally higher in spring than in summer (**Appendix S1**; **Supplementary Figure S17**; **Supplementary Table S3**). Both herbivory and pathogen damage changed with leaf chemistry mostly during summer, when leaf damage consistently increased with total phenolics in both the closed forest (**Appendix S1**; **Supplementary Figures S13, S14**) and the open woodland (**Appendix S1**; **Supplementary Figures S15, S16**). Leaf reserves and condensed tannins showed only weak associations with leaf damage (**Appendix S1**; **Supplementary Figures S13–S16**).

SEMs provided support to the findings obtained by the GLMMs. The proposed set of relationships revealed indirect associations between soil water content and leaf damage mediated by leaf defences (**Appendix S1**; **Supplementary Table S1**). During spring, only *Q. canariensis* trees showed bottom-up pathways (**Figure 2**), where soil water reduction was associated with higher leaf pathogen damage mediated by leaf defences (scenario 3; **Figure 1c**). During summer, total phenolics consistently mediated similar bottom-up associations between of soil water content and leaf damage by both herbivores and pathogens, across all species and sites (**Figure 3**).

**Figure 2:**
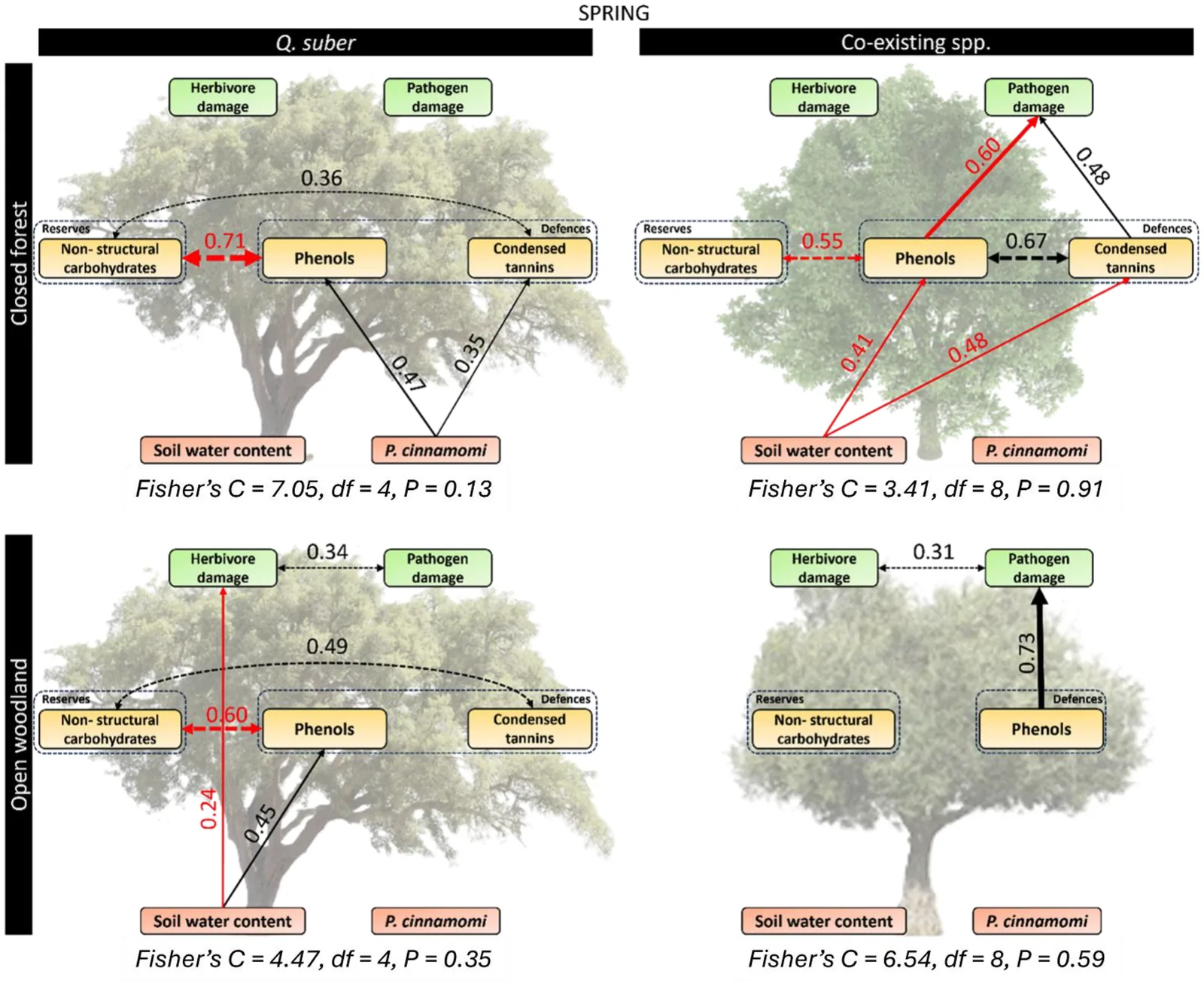
Structural equation models (SEMs) testing for cascading effects of soil water content and *P. cinnamomi* abundance on leaf chemistry (reserves and defences) and leaf damage by free feeding herbivores and fungal pathogens in spring. SEMs were run separately for each site and species combination (*Quercus suber* and *Q. canariensis* in the closed forest, *Q. suber* and *Olea europaea* in the open woodland). Values next to each arrow are standardized regression coefficients, where black and red values represent positive and negative associations, respectively. Continuous lines represent causal effects, and dashed lines represent correlations among variables.

**Figure 3:**
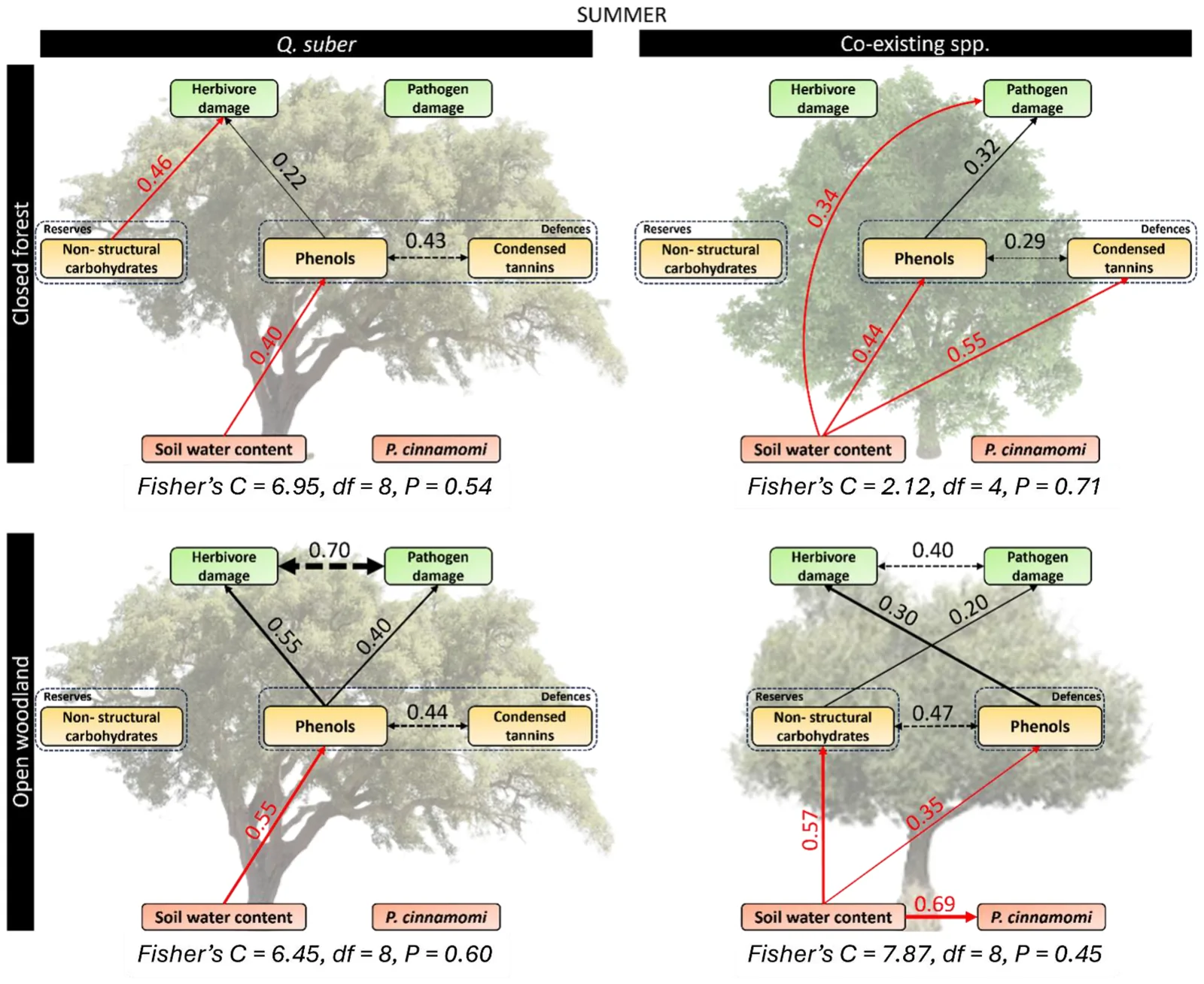
Structural equation models (SEMs) testing for cascading effects of soil water content and *P. cinnamomi* abundance on leaf chemistry (reserves and defences) and leaf damage by free feeding herbivores and fungal pathogens in summer. SEMs were run separately for each site and species combination (*Quercus suber* and *Q. canariensis* in the closed forest, *Q. suber* and *Olea europaea* in the open woodland). Values next to each arrow are standardized regression coefficients, where black and red values represent positive and negative associations, respectively. Continuous lines represent causal effects, and dashed lines represent correlations among variables.

## 4 Discussion

In this study, we aimed to advance our understanding of the cascading effects of drought on leaf chemistry and damage by antagonists (herbivores and pathogens) by combining a review of the existing literature with results from a field study simulating a drier climate in Mediterranean forests invaded by the exotic pathogen *Phytophthora cinnamomi*. The literature review showed that the number of studies testing the effects of soil water content on chemistry and damage aboveground remains limited. However, this review indicated a complex relationship among the three components (**Figure 1**), with most studies suggesting that higher damage levels are likely to occur under drier conditions. The results of our field study support the conclusion of increasing leaf damage with decreasing soil water content and suggest that this relationship is associated with changes in leaf total phenolics. Unlike soil water content, the soil-borne pathogen *P. cinnamomi* seldom triggered bottom-up effects on leaf damage and chemistry. Our findings provide valuable insights for predicting plant susceptibility to emergent pests and pathogens under future drought scenarios.

### 4.1 Leaf chemistry responses to reduced soil water content

Our study supports that variations in soil water content can translate into important changes in leaf chemistry, particularly regarding leaf defences during the dry season. We found that total phenolics in summer leaves increased significantly with decreasing soil water availability, but such effect was less consistent during spring. Mechanistically, this seasonal contrast might be related to shifts in plant phenology and environmental stress intensity across the growing season. During spring, high soil moisture and moderate temperatures generally support active photosynthesis and primary growth, potentially leading plants to prioritize structural development over heavy investment in secondary defences (Chapin et al., 1990; Herms and Mattson, 1992; Gaytán et al., 2022). Under these conditions, the demand for antioxidant protection may also be comparatively lower (Rivas‐Ubach et al., 2014). In contrast, summer drought combines high irradiance, elevated temperatures and reduced water availability, conditions that are often associated with increased oxidative stress and the accumulation of phenolic compounds involved in photoprotection and stress tolerance (Grace and Logan, 2000; Niinemets, 2010; Varela et al., 2016; Jansen et al., 2022; Park et al., 2023; Shoaib et al., 2023). Furthermore, while leaf reserves did not significantly change with soil water content, the increase in leaf total phenolics and condensed tannins under drier conditions may be compatible with shifts in resource allocation towards defensive compounds under drought stress (Hartmann and Trumbore, 2016). Leaf chemistry changes greatly throughout the growing season (Barton and Koricheva, 2010; Boege et al., 2011). Such ontogenetic variation might be one of the reasons behind the large variability of results found in the literature linking leaf chemistry and soil water content, since studies differed substantially in the time of measurement (**Table 1**). These studies highlight that leaf chemistry strongly depends on leaf age and plant maturation stage, potentially contributing to non-monotonic effects of drought on leaf chemistry (Gutbrodt et al., 2011, 2012a, Bisigato et al., 2015).

While soil water content significantly affected leaf chemistry in our Mediterranean forests across sites and species, we found little evidence for a relationship between the abundance of the soil-borne pathogen *P. cinnamomi* and leaf chemistry. Although previous studies have shown that soil pathogens can induce changes in plant chemistry that translate to aboveground tissues (Kaplan et al., 2018; Huberty et al., 2020), we suggest that such changes might be more likely detected in advanced stages of disease. In our study sites, the root rot disease driven by *P. cinnamomi* seems to be at an early stage given the low abundance of symptomatic trees (defoliation and dieback). Consequently, it is likely that pathogen-induced effects were more evident belowground than in leaf tissues (Homet et al., 2024). While our field study captured natural infection dynamics under realistic conditions, evaluating how increasing inoculum loads modulate host physiological responses will require future complementary experiments using controlled infection gradients.

### 4.2 Impact of induced changes in leaf chemistry on leaf damage

Our experimental results indicated that drought-induced increases in leaf chemical compounds were associated with higher levels of leaf damage, supporting scenario 3 (**Figure 1**). Specifically, phenolics were the main compounds mediating the indirect pathway of low soil water content on damage rates, linking drought-induced changes in leaf chemistry to plant-antagonist interactions. This positive association between phenolics and damage seems counterintuitive given the well-known defensive role of phenolics against antagonists (Hale et al., 2005; Kosonen et al., 2012; Kuczyk et al., 2021; Lin et al., 2021), as represented in scenario 2 (**Figure 1**). However, phenolic compounds can fulfil multiple ecological functions, and their effects on antagonists are not always negative. Indeed, some phenolics (e.g. quercetin) have been reported to stimulate herbivory and growth of fungal pathogens (Close and McArthur, 2002; Bais et al., 2010; Kosonen et al., 2012; Ahammed et al., 2023). In our experiment, this positive association became significantly apparent during summer, coinciding with peak phenolic concentrations and higher levels of leaf damage (Rivas‐Ubach et al., 2014). These findings suggest that the predicted increase in severity and frequency of droughts in Mediterranean ecosystems under climate change (Stocker et al., 2013; Ali et al., 2022) could be linked to higher leaf damage through shifts in leaf chemistry, highlighting a potential trade-off between drought tolerance and defence.

Among the case studies included in our literature review, only two (Prill et al., 2014; Rivas‐Ubach et al., 2014) showed a pattern similar to that observed in our field study, with low soil water availability associated with higher leaf defences and damage (scenario 3; **Figure 1**). Although there are fundamental differences with our study, they were also the only ones conducted under field conditions. Moreover, Rivas‐Ubach et al. (2014) was the only study exploring drought effects in a Mediterranean oak forest and its implications for leaf damage caused by the entire community of free feeding herbivores. We argue that results from studies conducted under controlled conditions and focused on a single herbivore species (i.e., those dominating literature) fundamentally differ from results obtained under more realistic and complex field conditions (Asbjornsen et al., 2018; Zvereva et al., 2024). Our field study, covering three tree species in two locations invaded by the exotic pathogen *P. cinnamomi* in multiple years and seasons, aimed to capture part of the complexity that characterized Mediterranean forests (Peñuelas et al., 2002; Newbold et al., 2020; Ali et al., 2022). However, further field studies in other systems are needed to improve our understanding of potential indirect effects of increasing drought on leaf damage. Such studies would contribute to unveil whether the trends observed in Mediterranean forests are generalizable or, on the contrary, represent a specific characteristic of these water-limited systems that could make them particularly vulnerable to pests and pathogens in a drier world.

Our empirical findings provide new field-based insights into the relationships between plant chemistry and leaf damage under drought. First, our results complement classical Plant-Quality (Awmack and Leather, 2002) and Bottom-up theories (Hunter and Price, 1992), which predict that variation in plant traits and chemistry can influence the performance of higher trophic levels. While traditional models often assume that secondary metabolites such as phenolics primarily function as direct anti-herbivore defences (Stamp, 2003), our results suggested that drought-induced phenolic accumulation is associated with higher rather than lower leaf damage. This pattern highlights the multifunctional nature of phenolic compounds and suggests that their ecological roles may vary depending on the interacting organisms and environmental context (Close and McArthur, 2002). Second, our findings are broadly consistent with the Stress-Induced Susceptibility Theory (White, 1984), which proposes that physiological changes induced by environmental stress can increase plant susceptibility to natural enemies. The positive association between drought-related chemical responses and leaf damage observed in our study supports this possibility under field conditions. Finally, our results are compatible with the idea that trade-offs in resource allocation shape plant responses to multiple stressors, as proposed by Growth-Defence (Herms and Mattson, 1992) and Carbon Allocation theories (Chapin et al., 1990). By combining a systematic literature review with a multi-species field experiment, our study helps address the critical scarcity of field-based evidence on how drought-associated variation in leaf chemistry relates to plant-antagonist interactions.

## 5 Conclusions

Our results suggest that leaf damage caused by both insects and foliar pathogens is likely to increase under lower soil water availability. This implies that climate change might not only reduce tree performance directly by increasing water limitation (Adams et al., 2015; Chambi-Legoas et al., 2020; Ogaya and Peñuelas, 2020), but also indirectly by altering leaf traits that enhance insect herbivory and pathogen infection. The identity of the specific leaf traits underlying this pattern is, however, uncertain. Here we focused on leaf chemistry (particularly leaf chemical defences and reserves) as potential key traits, given their well-known role in determining leaf palatability (Barton and Koricheva, 2010; Castagneyrol et al., 2018; Gaytán et al., 2022). Furthermore, we acknowledge that leaf age, seasonal environmental variation, and the cumulative duration of damage cannot be fully separated in our spring-summer comparison, since these observational associations do not establish a definitive causal direction (Gaytán, 2026b). However, patterns reported in the literature were highly variable, suggesting that other physiological and morphological leaf traits might also mediate the cascading effects of soil water content on leaf damage (Gutbrodt et al., 2012b; Ganthaler et al., 2023). Interestingly, the two field studies conducted in Mediterranean forests (Rivas‐Ubach et al., 2014 and this study) converged in identifying phenolics as key compounds enabling plants to cope with drought, but at the cost of increasing levels of leaf damage (Bais et al., 2010; Kosonen et al., 2012; Ahammed et al., 2023). This finding suggests that tree species might face a fundamental trade-off in protecting themselves against the increasing stress imposed by the abiotic (drought) and biotic (pests and pathogens) drivers of global change.

## Data Availability

The raw data supporting the conclusions of this article will be made available by the authors, without undue reservation.
