# Supplementary material for: Cascading effects of soil water content on leaf chemistry and damage: implications for plant susceptibility to pests and pathogens in a drier world
Source: Front Plant Sci. 2026 Oct 20;17:1914073. doi: 10.3389/fpls.2026.1914073 (PMC13727387; doi:10.3389/fpls.2026.1914073)
Supplement: Supplementary file 1 [file SupplementaryFile1.docx]

Supplementary Material

# 1 Supplementary Figures and Tables

## 1.1 Supplementary Figures

**Figure S1.** Flow diagram of study selection for the systematic review

**Figure S2.** Overview of the study area, where panel **a)** shows the location of Los Alcornocales Natural Park in the Iberian Peninsula, panel **b)** shows a closer view of the study site, and **c)** shows an image of the rainfall exclusion infrastructure installed in one the study sites (the closed forest).

**Figure S3.** A) Monthly precipitation in the study area during the study period; B) Predicted values of volumetric soil water content (%) corrected by soil texture at different soil depths (10, 20, 30 and 40 cm) in the closed forest. Vertical grey lines separate different years. Rainfall exclusion started in March 2016. [Adapted from Gaytán et al. 2024]

**Figure S4.** A) Monthly precipitation in the study area during the study period; B) Predicted values of volumetric soil water content (%) corrected by soil texture at different soil depths (10, 20, 30 and 40 cm) in the open woodland. Vertical grey lines separate different years. Rainfall exclusion started in March 2016. [Adapted from Gaytán et al. 2024]

**Figure S5.** Examples of leaves of **a)** *Quercus suber*, **b)** *Quercus canariensis* and **c)** *Olea europaea* damaged by free feeding herbivores and foliar pathogens. Free feeding herbivore damage can be observed in leaf borders commonly chewed by lepidoptera larvae. Foliar fungal damage can be observed in the necrotic patches with a distinct dark margin, small scattered black patches or greyish necroses with distinct reddish-brown edges commonly caused by fungi belonging to *Cystodendron*, *Dendromophoma*, *Discula*, *Lembosia* and *Spilocaea* genera.

**Figure S6.** *A priori* structural equation model showing the rationale behind the direct and indirect effects among soil water content, abundance of *P. cinnamomi* in the soil, leaf chemistry (reserves = non-structural carbohydrates, and defences = total phenolics and condensed tannins) and leaf damage by free feeding herbivores and foliar pathogens.

**Figure S7.** Differences in volumetric soil water content (average for the 0-40 cm soil profile) between rainfall exclusion and control plots in the closed forest and the open woodland during spring and summer. Boxplots indicate interquartile distribution of the raw data and red points indicate mean values in each category.

**Figure S8.** Relationships between soil water content and leaf chemistry (non-structural carbohydrates = NSC, total phenolics and condensed tannins) in the closed forest, separately for each tree species (*Quercus suber* and *Q. canariensis*) and season (spring and summer). Shown are regression lines with their associated confidence intervals (shaded area) for significant relationships and dashed regression lines for trends. R^2^ and P-values are shown for significant relationships (P <0.05) and trends (P<0.10). Each dot represents an individual tree under rainfall exclusion (brown) or in control plots (blue).

**Figure S9.** Relationships between soil water content and leaf chemistry (non-structural carbohydrates = NSC, total phenolics and condensed tannins) in the open woodland, separately for each tree species (*Quercus suber* and *Olea europaea*) and season (spring and summer). Shown are regression lines with their associated confidence intervals (shaded area) for significant relationships and dashed regression lines for trends. R^2^ and P-values are shown for significant relationships (P <0.05) and trends (P<0.10). Each dot represents an individual tree under rainfall exclusion (brown) or in control plots (blue). Note that *Olea* trees do not produce condensed tannins.

**Figure S10.** Differences in non-structural carbohydrates (NSC), total phenolics and condensed tannins between spring and summer leaves of the four site-tree species combinations (*Q. suber* and *Q. canariensis* in the closed forest, *Q. suber* and *O. europaea* in the open woodland). P-values are given only for significant differences. Circles and vertical lines represent means and standard errors, respectively. Note that *Olea* trees do not produce condensed tannins.

**Figure S11.** Relationships between *Phytophthora cinnamomi* abundance in the soil (CFU g^-1^, colony forming units per gram of soil) and leaf chemistry (non-structural carbohydrates = NSC, phenolics and condensed tannins) in the closed forest, separately for each tree species (*Quercus suber* and *Q. canariensis*) and season (spring and summer). Shown are regression lines with their associated confidence intervals (shaded area) for significant relationships and dashed regression lines for trends. R^2^ and P-values are shown for significant relationships (P <0.05) and trends (P<0.10). Each dot represents an individual tree under rainfall exclusion (brown) or in control plots (blue).

**Figure S12.** Relationships between *Phytophthora cinnamomi* abundance in the soil (CFU g^-1^, colony forming units per gram of soil) and leaf chemistry (non-structural carbohydrates = NSC, phenolics and condensed tannins) in the open woodland, separately for each tree species (*Quercus suber* and *O. europaea*) and season (spring and summer). Shown are regression lines with their associated confidence intervals (shaded area) for significant relationships and dashed regression lines for trends. R^2^ and P-values are shown for significant relationships (P <0.05) and trends (P<0.10). Each dot represents an individual tree under rainfall exclusion (brown) or in control plots (blue). Note that *Olea* trees do not produce condensed tannins.

**Figure S13.** Relationships between leaf chemistry (non-structural carbohydrates = NSC, phenolics and condensed tannins) and damage for *Quercus suber* trees in the closed forest, separately for each season (spring and summer). Shown are regression lines with their associated confidence intervals (shaded area) for significant relationships and dashed regression lines for trends. R^2^ and P-values are shown for significant relationships (P <0.05) and trends (P<0.10). Each dot represents an individual tree under rainfall exclusion (brown) or in control plots (blue).

**Figure S14.** Relationships between leaf chemistry (non-structural carbohydrates = NSC, phenolics and condensed tannins) and damage for *Quercus canariensis* trees in the closed forest, separately for each season (spring and summer). Shown are regression lines with their associated confidence intervals (shaded area) for significant relationships and dashed regression lines for trends. R^2^ and P-values are shown for significant relationships (P <0.05) and trends (P<0.10). Each dot represents an individual tree under rainfall exclusion (brown) or in control plots (blue).

**Figure S15.** Relationships between leaf chemistry (non-structural carbohydrates = NSC, phenolics and condensed tannins) and damage for *Quercus suber* trees in the open woodland, separately for each season (spring and summer). . Shown are regression lines with their associated confidence intervals (shaded area) for significant relationships and dashed regression lines for trends. R^2^ and P-values are shown for significant relationships (P <0.05) and trends (P<0.10). Each dot represents an individual tree under rainfall exclusion (brown) or in control plots (blue).

**Figure S16.** Relationships between leaf chemistry (non-structural carbohydrates = NSC and total phenolics) and damage for *Olea europaea* trees in the open woodland, separately for each season (spring and summer). Shown are regression lines with their associated confidence intervals (shaded area) for significant relationships and dashed regression lines for trends. R^2^ and P-values are shown for significant relationships (P <0.05) and trends (P<0.10). Each dot represents an individual tree under rainfall exclusion (brown) or in control plots (blue).

**Figure S17.** Differences in herbivory and pathogen damage (%) between spring and summer leaves in *Q. suber* and *Q. canariensis* trees within the closed forest and in *Q. suber* and *O. europaea* within the open woodland. P-values are given only for significant differences. Circles and vertical lines represent means and standard errors (based on the raw data), respectively.

## 1.2 Supplementary Tables

**Table S1.** Indirect impacts of soil water content on leaf damage by herbivores and pathogens due to changes in non-structural carbohydrates (NSC), total phenolics and condensed tannins. Shown are parameter estimates and confidence intervals (CI) of the significant paths in both spring (Fig. 1) and summer (Fig. 2).

| **Season** | **Site** | **Species** | **Path** | **Estimate** | **CI** | |
| --- | --- | --- | --- | --- | --- | --- |
| Spring | Closed forest | *Q. canariensis* | SWC – Phenolics – Pathogen damage | -0.17 | -0.33 | -0.16 |
| Spring | Closed forest | *Q. canariensis* | SWC – Tannins – Pathogen damage | 0.16 | 0.09 | 0.48 |
| Summer | Closed forest | *Q. suber* | SWC – Phenolics – Herbivore damage | 0.20 | 0.02 | 0.33 |
| Summer | Closed forest | *Q. canariensis* | SWC – Phenolics – Herbivore damage | 0.21 | 0.07 | 0.22 |
| Summer | Open woodland | *Q. suber* | SWC – Phenolics – Pathogen damage | 0.26 | 0.04 | 0.65 |
| Summer | Open woodland | *Q. suber* | SWC – Phenolics – Herbivore damage | 0.36 | 0.17 | 0.61 |
| Summer | Open woodland | *O. europaea* | SWC – NSC – Pathogen damage | 0.19 | 0.18 | 0.20 |
| Summer | Open woodland | *O. europaea* | SWC – Phenolics – Herbivore damage | 0.14 | 0.09 | 0.30 |

**Table S2.** Results of the Linear Mixed Models that test the differences in leaf reserves (non-structural carbohydrates) and defences (total phenolics and condensed tannins) between spring and summer at the two study sites separately for each tree species. Block was included as fixed factor to control for spatial autocorrelation in the experimental design, whereas tree identity was included as a random factor to control for repeated measurements. Shown are χ^2^-values, degrees of freedom (df) and associated P-values.

| **Site** | **Species** | **Response** | **Factors** | **χ^2^** | **df** | **P-value** |
| --- | --- | --- | --- | --- | --- | --- |
| Closed forest | *Q. suber* | NSC | Block  Season | 0.31  31.18 | 2  1 | 0.859  <0.001 |
| Closed forest | *Q. suber* | Phenolics | Block  Season | 2.98  12.66 | 2  1 | 0.226  <0.001 |
| Closed forest | *Q. suber* | Tannins | Block  Season | 0.79  7.53 | 2  1 | 0.673  0.006 |
| Closed forest | *Q. canariensis* | NSC | Block  Season | 5.65  21.22 | 2  1 | 0.059  <0.001 |
| Closed forest | *Q. canariensis* | Phenolics | Block  Season | 13.24  14.41 | 2  1 | 0.001  <0.001 |
| Closed forest | *Q. canariensis* | Tannins | Block  Season | 6.72  14.03 | 2  1 | 0.035  <0.001 |
| Open woodland | *Q. suber* | NSC | Block  Season | 0.81  48.58 | 2  1 | 0.666  <0.001 |
| Open woodland | *Q. suber* | Phenolics | Block  Season | 5.78  13.98 | 2  1 | 0.056  <0.001 |
| Open woodland | *Q. suber* | Tannins | Block  Season | 14.03  0.46 | 2  1 | <0.001  0.498 |
| Open woodland | *O. europaea* | NSC | Block  Season | 1.25  51.80 | 2  1 | 0.536  <0.001 |
| Open woodland | *O. europaea* | Phenolics | Block  Season | 6.96  2.38 | 2  1 | 0.031  0.123 |

**Table S3.** Results of the Linear Mixed Models that test the differences in herbivore and pathogen damage between spring and summer at the two study sites separately for each tree species. Block was included as fixed factor to control for spatial autocorrelation in the experimental design, whereas tree identity was included as a random factor to control for repeated measurements. Shown are χ^2^-values, degrees of freedom (df) and associated P-values.

| **Site** | **Species** | **Response** | **Factors** | **χ^2^** | **df** | **P-value** |
| --- | --- | --- | --- | --- | --- | --- |
| Closed forest | *Q. suber* | Herbivore damage | Block  Season | 3.52  0.26 | 2  1 | 0.172  0.613 |
| Closed forest | *Q. suber* | Pathogen damage | Block  Season | 3.25  15.29 | 2  1 | 0.197  <0.001 |
| Closed forest | *Q. canariensis* | Herbivore damage | Block  Season | 0.97  1.00 | 2  1 | 0.616  0.316 |
| Closed forest | *Q. canariensis* | Pathogen damage | Block  Season | 4.54  150.88 | 2  1 | 0.104  <0.001 |
| Open woodland | *Q. suber* | Herbivore damage | Block  Season | 3.40  4.35 | 2  1 | 0.182  0.037 |
| Open woodland | *Q. suber* | Pathogen damage | Block  Season | 0.72  12.58 | 2  1 | 0.697  <0.001 |
| Open woodland | *O. europaea* | Herbivore damage | Block  Season | 3.21  2.08 | 2  1 | 0.201  0.150 |
| Open woodland | *O. europaea* | Pathogen damage | Block  Season | 3.37  0.06 | 2  1 | 0.186  0.805 |
